# High prevalence of medication errors in a secondary‐level Lithuanian hospital: A prospective cross‐sectional observational study

**DOI:** 10.1002/prp2.1246

**Published:** 2024-07-31

**Authors:** J. Butauskaite, A. Zumbakyte, L. Aukstikalne, J. Pancere, S. Zukaitiene, E. Karinauske

**Affiliations:** ^1^ Medical Academy, Faculty of Medicine Lithuanian University of Health Sciences Kaunas Lithuania; ^2^ Medical Academy, Faculty of Medicine, Institute of Physiology and Pharmacology Lithuanian University of Health Sciences Kaunas Lithuania

**Keywords:** clinical pharmacology, drug monitoring, inpatient care, polypharmacy, medication errors, medication education

## Abstract

As the population continues to age, the occurrence of chronic illnesses and comorbidities that often necessitate the use of polypharmacy has been on the rise. Polypharmacy, among other factors that tend to coincide with chronic diseases, such as obesity, impaired kidney and liver function, and older age, can increase the risk of medication errors (MEs). Our study aims to evaluate the prevalence of MEs in the Internal medicine, Cardiology, and Neurology departments at the secondary‐level university hospital. We conducted a prospective observational study of 145 patients’ electronic or paper‐based data of inpatient prescriptions and patients' pharmacokinetic risk factors, such as an impairment of renal and/or hepatic function, weight, and age. All included patients collectively received 1252 prescribed drugs. The median (Q1; Q3) number of drugs per patient was 8 (7;10). At least one ME was identified in 133 out of the 145 patients, indicating a significantly higher prevalence than hypothesized (91.7% vs. 50%; *p* < .001). There was moderate, positive correlation between the quantity of prescribed drugs and the number of MEs, meaning that the more drugs are prescribed, the higher the number of identified MEs (Spearman's ρ = 0.428; *p* < .001). These findings suggest that there is a need for continuous medication education activity for prescribing physicians, continuous evaluation of prescription appropriateness to objectively identify the MEs and to contribute to more rational patient treatment.

**Abbreviations**
BMI: body mass index
LBM: lean body mass
LUHS: lithuanian university of health sciences
MD: medical doctor
ME: medication error
SmPC: Summary of Product Characteristics

## 1 INTRODUCTION

As the population continues to age, the occurrence of chronic illnesses and comorbidities that often necessitate the use of polypharmacy has been on the rise.^1^, ^2^ Polypharmacy, among other factors that tend to coincide with chronic diseases such as obesity, impaired kidney and liver function, and older age, can increase the risk of medication errors (MEs). The US National Coordinating Council for Medication Error Reporting and Prevention proposes the following definition for MEs: “A medication error is any preventable event that might result in inappropriate medication use or patient harm while the medication is under the control of healthcare professionals, patients, or consumers. These events can be associated with various aspects of professional practice, healthcare products, procedures, and systems, encompassing prescribing, order communication, product labelling, packaging, and nomenclature, compounding, dispensing, distribution, administration, education, monitoring, and use”.^3^ The prevalence of MEs varies from 18% to more than 40% across different healthcare settings.^4^ Although some studies suggest that the prevalence of MEs may even reach as high as 79%.^5^

In Lithuania, research on MEs has been conducted in tertiary care medical wards. A study published in 2017 revealed staggering rates of MEs—prescription errors were observed in 49% of cases analyzed.^6^ Furthermore, a small sample size cross‐sectional pilot study conducted at the Department of Psychiatry in Lithuanian University of Health Sciences (LUHS) Hospital Kaunas Clinics in 2019 analyzed the data of 33 patients, 29 (87.9%) of whom presented with at least one case of MEs.^7^ Previous studies also show a lack of knowledge on over‐the‐counter medications among patients.^8^ Our study aims to evaluate the prevalence of MEs in the internal medicine, cardiology, and neurology departments at the secondary‐level university hospital. We hope that the results help create strategies to provide safe and efficient treatment to patients and reduce medication‐related spending for hospitals.

## 2 METHODS

### 2.1 Design, setting, and population

The study was approved by the Kaunas Regional Biomedical Research Ethics Committee (No. BE‐2‐84). This was a prospective cross‐sectional observational study carried out between June 1, 2021 and May 31, 2023 at the Departments of Neurology, Cardiology, and Internal Medicine at the secondary‐level university hospital. Patient population and sample size were calculated using official numbers of discharged patients published by these departments in 2019–2020. The number of admitted patients dropped in 2020 due to COVID‐19 restrictions, however, considering a likely increase over the course of the study, patient population, and sample size were calculated using averages from 2019 to 2020: 6027 patients in 2019 and 3883 patients in 2020, bringing the average to 4955. For the study to reach 95% statistical significance with an alpha value of .05 and a population of 4955, the sample size should be not smaller than 135.

Study subjects were chosen using the following inclusion criteria:

- Consent to participate in the study
- Admission to one of the medical wards (internal medicine, cardiology, or neurology)
- Adult patients (≥18 years of age)
- Patients receiving two or more medications

Withdrawn using the following exclusion criteria:

- Refusal to participate
- Receiving one medication only
- Receiving no medications

### 2.2 Data collection

Patient data were collected using paper‐based and electronic medical records throughout inpatient treatment. The following data were colleced during the study: patient age, gender, weight, height, lean body mass (LBM), adjusted body mass, body mass index (BMI), plasma creatinine levels, the main reason for hospital admission, medical history, prescribed medications with indications, dosage, start and end dates of administration, information on whether medication safety and efficacy were monitored; adverse drug reactions, related laboratory and instrumental test data, outcomes.

When reviewing a patient's prescriptions, the following were considered: conformity to indications/contraindications, dosage, contraindications listed in the summary of product characteristics (SmPC) or official treatment guidelines, and national/international drug databases such as Micromedex or UpToDate. Kidney function was evaluated by calculating creatinine clearance with the Cockroft‐Gault formula unless otherwise stated in the SmPC. For obese patients, lean body mass was used in these calculations, unless otherwise stated. Creatinine clearance was calculated using LBM when BMI was ≥30 kg/m^2^. For patients with hepatic impairment, hepatic function was evaluated by calculating Child–Pugh score.

### 2.3 Screening for potential medication errors

Prescriptions were evaluated by the two independent investigators, if there were disagreements regarding the evaluation, the final decision was made by the third investigator. The reviewers were either a clinical pharmacologist, MD or a resident physician in clinical pharmacology program. A prescription was considered safe in the absence of contraindications or adverse effects, if the necessary tests were conducted to ensure drug safety, and the dosage was adjusted based on patient‐specific factors such as age, weight, and kidney and liver function. The effectiveness of a drug was determined by adherence to registered indications and dosage guidelines, coupled with the achievement of the desired clinical effect, which could include the reduction or disappearance of symptoms and positive changes in biomarkers. Moreover, cost‐effectiveness was established if a drug was prescribed in an appropriate dose and proved to be the least costly among pharmaceutical alternatives meeting the same standards of effectiveness and safety. Appropriate monitoring was defined as continuous safety and efficacy assessments through relevant testing. Finally, prescription rationality was evaluated by considering all the characteristics, including safety, efficacy, monitoring, and cost‐effectiveness. Examples of MEs recorded in our study included but were not limited to, off‐label use, deviations from recommended dosage, disregard of contraindications, clinically significant drug–drug interactions, and adverse drug reactions requiring treatment or monitoring.

### 2.4 Statistical analysis

Descriptive statistics were employed to characterize both the included patient sample and their respective prescriptions. Nominal and categorical variables were presented using frequency tables. Interval variables (such as age, creatinine clearance, and weight) underwent normality testing. Normally distributed variables were summarized using mean and standard deviation, whereas nonnormally distributed variables were described using median, Q1, Q3, and, where necessary, minimum, and maximum values.

Binominal test was used to evaluate the observed prevalence of MEs compared to the hypothesized 90%. Spearman's correlation was run to determine the relationship between the number of MEs and interval variables, including weight, creatinine clearance, age, and the number of drugs prescribed. To investigate the relationship between ME (none vs. at least one) and categorical variables such as gender (female vs. male), renal function (normal/mild vs. moderate to severe vs. hyperfiltration), hepatic function (normal vs. impaired), and BMI categories (under 25 vs. 25 and more), the chi‐square test was utilized. For assessing the influence of potential factors on the presence of at least one ME, binomial logistic regression was initially planned. However, due to the exceptionally high prevalence of at least one ME, the regression model did not fit the data and could not be validly used.

The significance level (α) for hypothesis tests was set at .05. Statistical analyses were performed using SPSS v29.0.1.0 software.

## 3 RESULTS

One hundred and forty‐five patients were enrolled in the study, and they collectively received 1252 prescribed drugs. The median (Q1; Q3) number of drugs per patient was 8 (7;10). Notably, at least one ME was identified in 133 out of the 145 patients, indicating a significantly higher prevalence than hypothesized (91.7% vs. 50%; *p* < .001). It is worth mentioning that six patients (4.1%) exhibited at least one ME across all assessed categories. Table 1 outlines the characteristics of the patients.

**TABLE 1:** Patient characteristics.

*N* = 145		Frequency	Percent
Age, years (median; (Q1; Q3))	71 (61;81)		
Drugs, number (median (Q1; Q3))	8 (7;10)		
Sex	F	90	62.1
	M	55	37.9
BMI, kg/m^2^ (mean (SD))	28.8 (5.4)		
BMI category	<25	34	23.4
	25–30	53	36.6
	30–35	38	26.2
	35–40	15	10.3
	>40	5	3.4
Creatinine clearance (Cockcroft‐Gault), mL/min (median (Q1;Q3))	71 (49;97)		
Renal function	Normal	29	20.0
	Mild	55	37.9
	Moderate	43	29.7
	Severe	5	3.4
	Renal replacement therapy	0	0
	Hyperfiltration	11	7.6
	Missing data	2	1.4
Hepatic function	Normal	141	97.2
	Child–Pugh A	0	0
	Child–Pugh B	2	1.4
	Child–Pugh C	2	1.4

The assessment of various MEs revealed noteworthy findings. The median number of any ME was 11 (Q1: 4.5; Q3: 16.5), ranging from 0 to 35. Predominant among the identified ME was a lack of cost‐effectiveness. A comprehensive breakdown of all ME categories, along with their respective medians (Q1; Q3) per patient, is provided in Table 2.

**TABLE 2:** The median (Q1;Q3) number of specific medication errors (MEs) per patient.

	Median (Q1;Q3)
Any kind of ME	11 (4, 5;16, 5) (min 0;max 35)
Off‐label use (the median (Q1;Q3) number of off‐label drugs prescribed per patient)	2 (1;3)
Inappropriate dosing/dosing regimen	1 (0;1)
Contraindicated drugs	0 (0;0)
Lack of efficacy monitoring	1 (0;2)
Lack of safety monitoring	0 (0;2)
Not cost‐effective	3 (1;4)
Irrational	3 (1;4,5)

**FIGURE 1:**
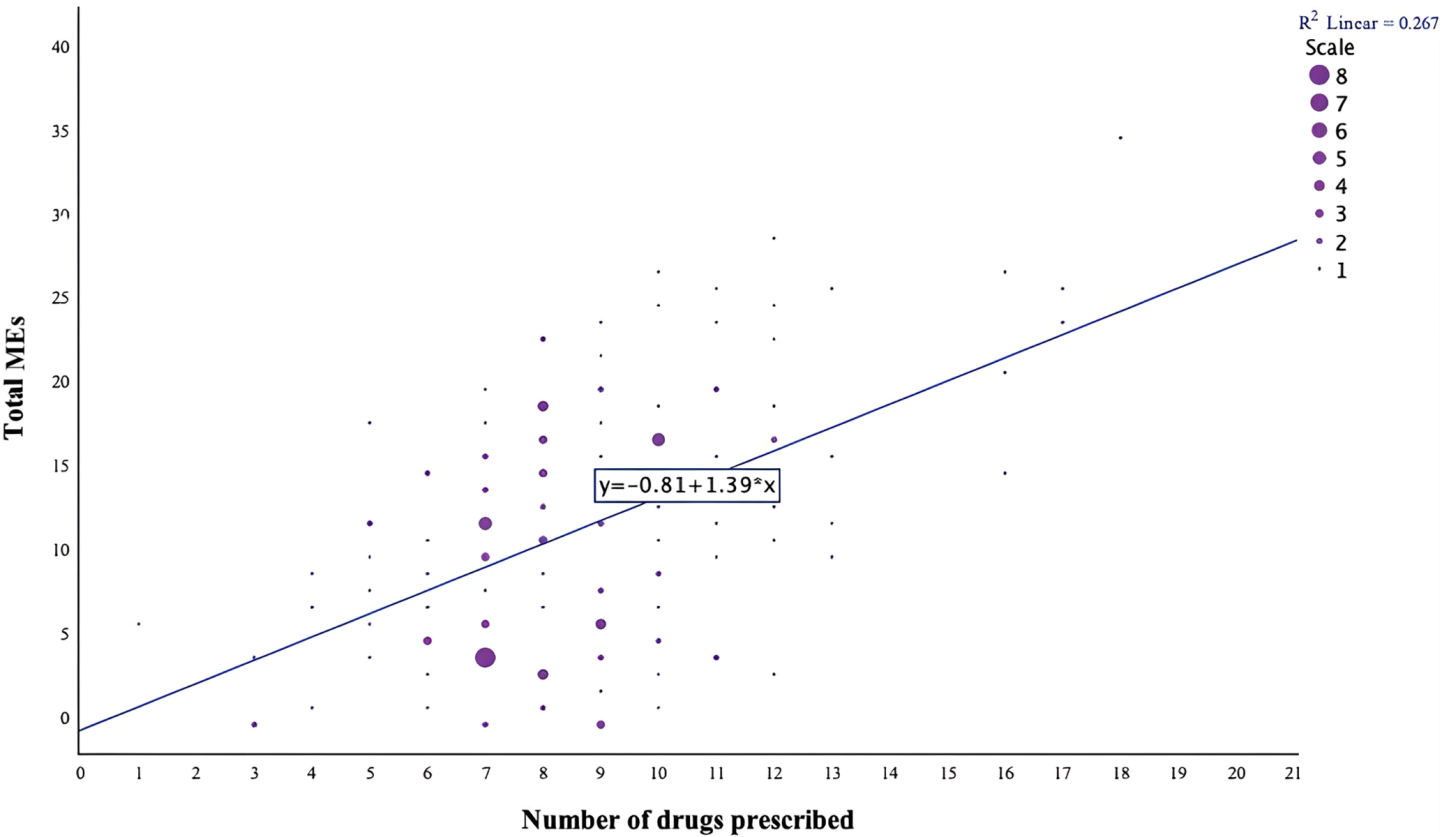
Correlation between the quantity of prescribed drugs and the number of medication errors (Spearman's ρ = 0.428; *p* < .001).

There was a moderate, positive correlation between the quantity of prescribed drugs and the number of MEs, meaning that the more drugs are prescribed, the higher the number of identified MEs (Spearman's ρ = 0.428; *p* < .001) (Figure 1). However, there was no correlation observed between the number of MEs and age, weight, or creatinine clearance (*p* > .05). Moreover, there was no significant relationship between the presence of MEs and gender, renal function, hepatic function, or BMI (*p* > .05).

**FIGURE 2:**
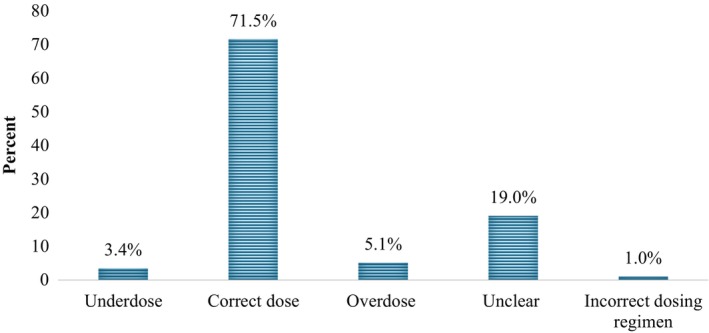
Dosing variations of prescribed drugs.

Additionally, drug‐level analysis was undertaken, encompassing a total of 1252 prescribed drugs for 145 patients, as previously mentioned. The assessment of overall rationality revealed that 35.8% (448 drugs) were classified as not meeting rationality criteria. Notably, contraindications were identified in 2.3% (29 drugs). Regarding efficacy monitoring, a substantial 83.9% (1051 drugs) adhered to monitoring practices, whereas 12.3% (154 drugs) did not, and 3.8% (47 drugs) were considered unclear due to lack of data. Variability in dosing adequacy was observed, with 3.4% (43 drugs) being underdosed, 71.5% (895 drugs) correctly dosed, 5.1% (64 drugs) exhibiting overdose, 19.0% (238 drugs) remaining unclear, and 3.4% (12 drugs) displaying an incorrect dosing regimen (Figure 2). These findings, along with results from other rationality domains, are presented in Table 3.

**TABLE 3:** Drug‐level analysis of medication errors.

*N* = 1252	Frequency	Percent
Overall rationality		
Yes	804	64.2
No	448	35.8
Contraindications		
Yes	29	2.3
No	1214	97.0
Lack of data to assess	9	0.7
Efficacy monitoring		
Yes	1051	83.9
No	154	12.3
Lack of data to assess	47	3.8
Safety monitoring		
Yes	935	74.7
No	134	10.7
Lack of data to assess	183	14.6
Pharmacoeconomics		
Yes	818	65.3
No	427	34.1
Lack of data to assess	7	0.6
On label use		
Yes	975	77.9
No	277	22.1
Dosing adequacy		
Underdose	43	3.4
Correct	895	71.5
Overdose	64	5.1
Unclear	238	19.0
Incorrect dosing regimen	12	3.4

## 4 DISCUSSION

This study, involving 145 patients and encompassing the prescription analysis of 1252 drugs, revealed several significant insights into MEs and rationality issues in pharmacotherapy. Remarkably, 91.7% of patients were exposed to at least one ME, exceeding both the hypothesized 50% and ME prevalence rates reported in other studies.^4^, ^5^

The median number of drugs per patient per day was 8, a well‐established factor associated with an increased likelihood of encountering MEs.^4^ Confirming this, a moderate positive correlation was identified between the quantity of prescribed drugs and the occurrence of MEs. However, despite this correlation, no significant relationships were observed between the presence of MEs and patient‐specific factors such as age, weight, sex, renal function, hepatic function, or BMI. This suggests that MEs are more influenced by the complexity and volume of the drug regimen than specific patient characteristics.

The detailed assessment of MEs highlighted cost‐effectiveness issues as particularly prevalent, underscoring the need to consider economic factors to enhance resource utilization. Further drug‐level analysis revealed that a substantial proportion (35.8%) of prescribed drugs did not meet rationality criteria, with 2.3% being contraindicated. Additionally, 8.5% of drugs had inadequate dosing, including 5.1% being overdosed. While efficacy and safety monitoring were satisfactory in 83.9% and 74.7% of cases, respectively, 12.3% and 10.7% lacked adequate monitoring, emphasizing the necessity for consistent oversight to ensure therapeutic effectiveness.

These findings emphasize the importance of comprehensive medication management strategies, including thorough prescription reviews, continuous monitoring, and adherence to rational prescribing practices. Implementing interventions targeting ME reduction, enhancing cost‐effectiveness, and ensuring dosing adequacy could significantly enhance the quality of patient care and resource management.

## 5 CONCLUSIONS

The primary strength of this study lies in its execution across diverse hospital departments, providing a varied patient population that enhances the applicability of the findings. The main limitation of this study is that we did not gather data on the clinical outcomes and cannot show if the number of MEs has any impact on patient's health. The observational design, while suitable for determining the prevalence of MEs, suggests that this study serves as preliminary groundwork for more substantial investigations, particularly in the light of the noteworthy results it has yielded.

Our findings show that there are a lot of MEs in the therapeutic departments of a secondary hospital: the number of medications correlated with the number of MEs identified. These findings suggest that there is a need for continuous medication education activity for prescribing physicians, continuous evaluation of prescription appropriateness to objectively identify the MEs and to contribute to more rational patient treatment.

## AUTHOR CONTRIBUTIONS

EK and LA planned the study—developed a protocol and other documentation mandatory to get the permission from the Bioethics Committee to start the study. JB, AZ, and EK collected the informed consent forms and if the patient was eligible for inclusion in the study collected paper‐based and electronic medical records. LA, EK, and JP assessed all the drug prescriptions and summarized the findings. SZ carried out the statistical analysis. All authors contributed to the writing of the paper.

## FUNDING INFORMATION

No funding was received for this study.

## CONFLICT OF INTEREST STATEMENT

All authors declare that they have no conflicts of interest.

## ETHICS STATEMENT

The study was approved by the Kaunas Regional Biomedical Research Ethics Committee (No. BE‐2‐84) and the Informed consent forms from the patients were collected and are stored at the PI’s office.

## Data Availability

The data that support the findings of this study are available from the corresponding author upon reasonable request.
